# Computational modeling of the olfactory receptor Olfr73 suggests a molecular basis for low potency of olfactory receptor-activating compounds

**DOI:** 10.1038/s42003-019-0384-8

**Published:** 2019-04-23

**Authors:** Shuguang Yuan, Thamani Dahoun, Marc Brugarolas, Horst Pick, Slawomir Filipek, Horst Vogel

**Affiliations:** 1 Shenzhen Institutes of Advanced Technology, Chinese Academy of Sciences, Shenzhen, 518055 China; 2 Institute of Chemical Sciences and Engineering, Ecole Polytechnique Fédérale de Lausanne (EPFL), CH-1015 Lausanne, Switzerland; 3 Laboratory of Biomodelling, Faculty of Chemistry & Biological and Chemical Research Centre, Uni-versity of Warsaw, 02-093 Warsaw, Poland

**Keywords:** Computational biophysics, Virtual drug screening, Drug screening, Protein function predictions

## Abstract

The mammalian olfactory system uses hundreds of specialized G-protein-coupled olfactory receptors (ORs) to discriminate a nearly unlimited number of odorants. Cognate agonists of most ORs have not yet been identified and potential non-olfactory processes mediated by ORs are unknown. Here, we used molecular modeling, fingerprint interaction analysis and molecular dynamics simulations to show that the binding pocket of the prototypical olfactory receptor Olfr73 is smaller, but more flexible, than binding pockets of typical non-olfactory G-protein-coupled receptors. We extended our modeling to virtual screening of a library of 1.6 million compounds against Olfr73. Our screen predicted 25 Olfr73 agonists beyond traditional odorants, of which 17 compounds, some with therapeutic potential, were validated in cell-based assays. Our modeling suggests a molecular basis for reduced interaction contacts between an odorant and its OR and thus the typical low potency of OR-activating compounds. These results provide a proof-of-principle for identifying novel therapeutic OR agonists.

## Abstract (web summary)

Shuguang Yuan et al. report molecular modeling of the prototypical olfactory receptor Olfr73 and find that the binding pocket is much smaller but more flexible than that of typical G-protein-coupled receptors. They virtually screen 1.6 million compounds and predict 25 Olfr73 agonists, 17 of which are validated by cell-based assays.

## Introduction

The mammalian olfactory system has the intriguing capability of sensing and distinguishing a huge variety of chemical compounds, often with a considerable sensitivity and specificity^1–3^. The perception of smell of volatile molecules starts with the activation of olfactory receptors (ORs), translating the environmental chemical signals with the involvement of heterotrimeric G proteins into neuronal electrical responses. ORs are assumed to fold into a seven-transmembrane (7TM) helix structure establishing the largest class of G-protein-coupled receptors (GPCRs)^2^. More than 1000 ORs in mice and about 350 ORs in humans are dedicated to smell^3^, capable of detecting an enormous repertoire of chemical compounds by a combinatorial coding scheme. Typically, one OR recognizes multiple odorants and one odorant is recognized by multiple ORs, but different odorants are recognized by different combinations of ORs^1,3,4^. Although some progress in functional identification of ligands for ORs has been achieved in recent years, most ORs are orphan receptors, whose ligand repertoires remain to be determined^5–8^. This, together with the lack of high-resolution 3D structures of ORs, is the major reason why the fundamental mechanistic principles of odorant recognition and discrimination by ORs are largely unresolved at a molecular level. This is in contrast to non-olfactory GPCRs for which an increasing number of high-resolution 3D structures have fostered understanding of ligand/receptor interactions, of ligand-mediated transmembrane signaling, and of how they serve as critical templates for rational drug design^9^.

In the case of missing experimental structural and functional data, computer modeling can deliver reliable propositions on ligand structures, which may interact and activate a specific GPCR^10–14^. As ORs share similarities with the structure and function of class A GPCRs^15^, known structures of class A GPCRs have been used as templates for homology modelling of ORs. Combined with functional data on odorant-induced activation of wild-type and mutant ORs, computer modelling have generated reliable propositions for the structure of the ligand-binding sites and specific ligand-binding modes of particular ORs thereby gaining insight into odorant selectivity^16–21^.

The large number of ORs and their promiscuity in ligand-binding related to the combinatorial code opens a huge chemical range of OR specific agonists and antagonists^7,22,23^. Estimates under discussion range from thousands to billions of OR-activating compounds^1,3,24–26^. Decoding the large odorant library would be of interest for several reasons. First, to understand the fundamental principles of the combinatorial code of ligand binding to ORs, and how ligand binding eventually activates or suppresses signal transmission to the intracellular side of a particular olfactory receptor and finally induce neuronal responses. Second, to understand better olfaction related diseases and to find novel therapeutic strategies for their treatment. As an example, the correlation between the appearance of neurodegenerative diseases and dysfunction of the olfactory system is well known^27^. Interestingly, ORs are expressed in many non-olfactory tissues including, testis, tongue, heart, spleen, pancreas, lung, kidney and placenta^28,29^. This suggests that ORs influence non-olfactory processes and there are indications for that in sperm chemotaxis^30^, embryonic development and cell–cell recognition^31^, chemosensory functions in kidney^32^, proliferation of cancer cells^33,34^, and other metabolic processes intimately linked with the endocrine systems^35^ that regulate the bodies energy balance, revealing a connection between the appearance of diabetes and the dysfunction of the olfactory system^36–38^. To date mostly synthetic odorants have been identified to activate ectopically expressed ORs, for instance, in wound healing processes^39^ or by influencing gut motility through OR activation and the release of serotonin in human gastrointestinal enterochromaffin cells^40^. In pathologic processes synthetic odorant molecules were shown to influence different stages of cancer development^41,42^. More recent findings suggest that ORs might be targetable not only by classical odorant molecules but even by therapeutic compounds. The anesthetic drug ketamine, which elicits various neuropharmacological effects, including sedation, analgesia, and anti-depressant activity, has been shown to activate odorant receptors in olfactory sensory neurons and in the mouse brain^43^. In the present study we find that Olfr73 can also be a target of drug-like molecules. Such findings are important to unravel the poly-pharmacology of therapeutic agents interacting with multiple intended but also unintended targets^44^.

On the other hand, OR-activating compounds have been found to also activate non-olfactory receptors^45^. A better understanding of the odorant-OR structure–activity relationship will have impact beyond medical applications, including production of fragrances, perfumes, food and beverages, and offering new ways of rodent and non-insect pest control, to mention a few^7,46^.

**Fig. 1:**
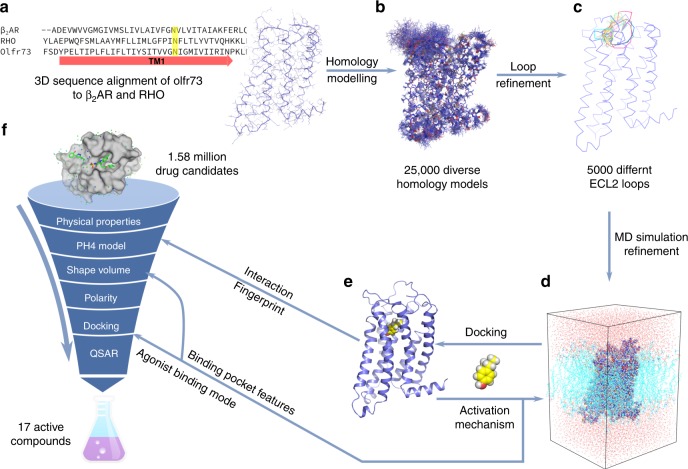
Workflow of virtual screening for new agonists of Olfr73. **a** Homology modelling of Olfr73 based on 3D structure-sequence alignment of Olfr73 to β_2_AR and Rho. **b** Refinement of ECL2 loop. **c** Molecular dynamics (MD) simulations of agonist bound Olfr73. **d** Docking agonist molecule into the 3D structural model of Olfr73. **e** Interaction fingerprint analysis by docking 25 reported compounds. This information was subsequently used for PH4 model building in virtual screening. **f** Virtual screening for Olfr73 agonists in the ZINC compound library composed of 1.58 million drug candidates. Applying stepwise selection filtering based on shape volume, ionization penalty and polarity, downscaled the chemical library successively to 204 compounds. The shape volume features are deduced from the results of MD simulations. Finally, quantitative structure–activity relationship evaluations reduced the chemical library to 64 compounds with predicted potential to activate Olfr73. Out of this final list, agonist binding modes were verified manually based on the activation mechanism deduced from MD simulations, and 25 available compounds were tested by cellular functional assays yielding 17 active compounds

Here we report on a computer-directed structure-function study to discover fundamental principles of how odorants bind to a prototypical olfactory receptor and to find subsequently new receptor-activating compounds beyond traditional chemical odorant libraries. For our study, we have chosen the mouse eugenol olfactory receptor Olfr73, as this receptor has been functionally expressed in heterologous mammalian cells for compound screening^16,17,47^. Figure 1 outlines the approach of our study. First, we built a homology model of the structure of Olfr73 and refined it by molecular dynamics simulations. Second, we used ligand docking to identify interaction fingerprints of various agonists in the receptor’s binding pocket, which together with molecular dynamics simulations of the agonist-receptor complexes revealed structural details on the first steps of receptor activation following ligand binding. Finally, the structural models served as a reliable base to perform a virtual computer screening of the ZINC library of 1.6 million drug-like molecules. The in silico screen selected from the large compound library considerably reduced the number of potential OR-activating compounds out of which a high percentage showed functional activity in cell-based assays. This result underscores the capacity of our in silico approach for rapid identification of potentially activating compounds out of large chemical libraries. Combining the 25 already known agonists with the 17 newly identified compounds establishes one of the largest collections of active compounds for a specific OR from which substantially improved conclusions on the characteristics of ligand–OR interactions can be extracted.

## Results

### Homology models of Olfr73

To obtain a reliable 3D model of Olfr73 through the initial homology modelling, we first compared the sequence of Olfr73 with sequences of other class A GPCRs in the PDB database. We found that it shared in the best case 19% sequence identity with beta-2-adrenergic receptor (β_2_AR, pdb code: 4LDE)^48^ and 16% sequence identity with rhodopsin (RHO, pdb code: 4BEY)^49^. Since multiple modelling templates can considerably improve the reliability of homology models^50–52^, we used the crystal structures of both receptors as templates for model building. 3D sequence alignment (Supplementary Fig. 1) indicates that most highly conserved residues/motifs in class A GPCRs^9^ including N^1.50^, D^2.50^, DRY motif, W^4.50^, Y^5.58^, F^6.44^ and NPxxY motif are also present in Olfr73^53^. However, residue P^5.50^ and motif CWxP that are typically found in non-OR class A GPCRs are missing in Olfr73. Moreover, one gap in TM3 is observed between the sequence of C^3.25^ and DRY motif of Olfr73 (Supplementary Fig. 1).

### Interaction fingerprints (IFP) between agonists and Olfr73

**Fig. 2:**
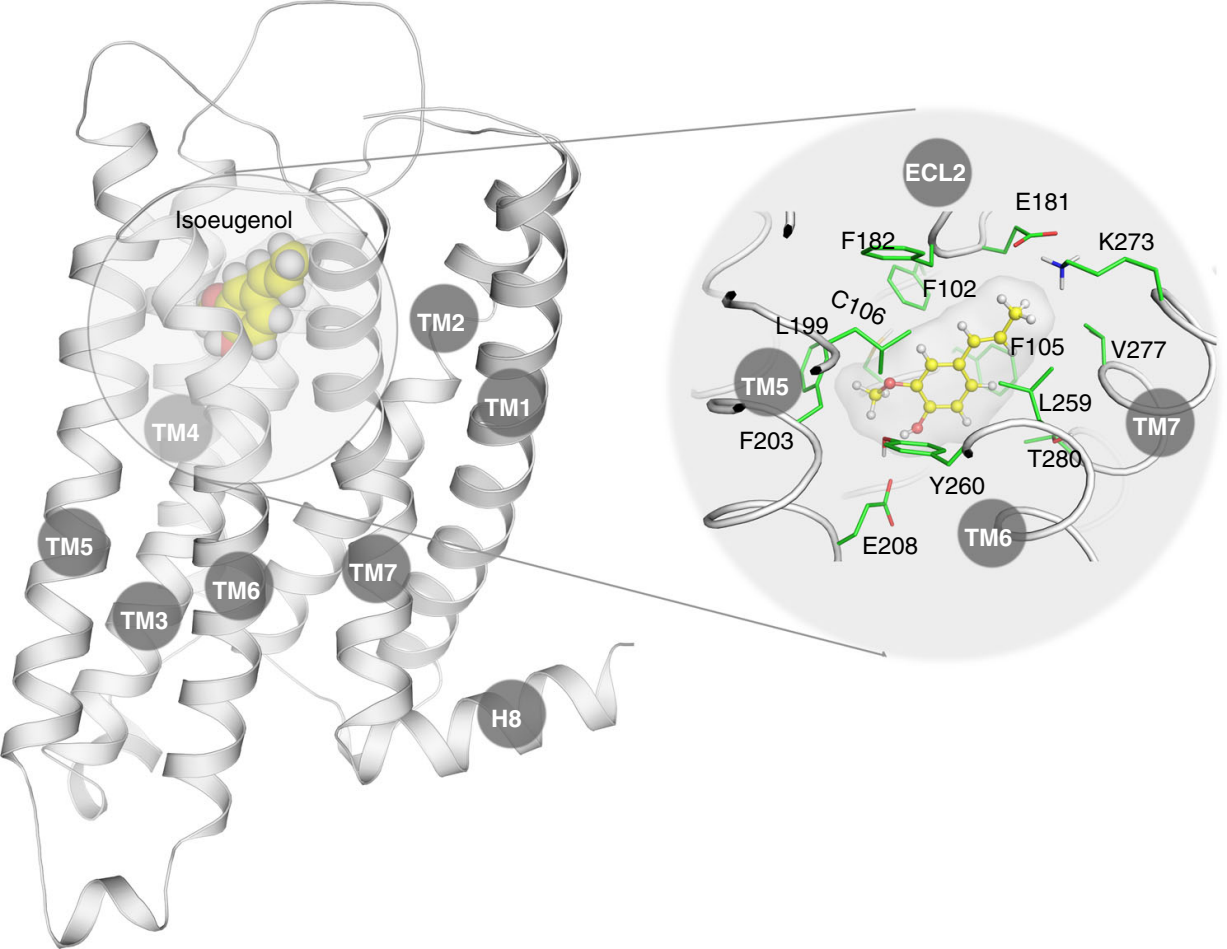
The 3D structural model of Olfr73 (left) and enlarged view of the binding mode of the agonist isoeugenol (right). Amino acid side chains in contact with bound isoeugenol are shown in green

The final refined homology model of Olfr73 (Fig. 2) shared many of the common features of non-OR class A GPCRs outlined elsewhere^9,54^. We then docked isoeugenol, a potent agonist for Olfr73^47^, into the predicted extracellular ligand-binding pocket to explore atomistic details of the interaction between the ligand and its receptor. As depicted in Fig. 2, the hydrophobic moiety of isoeugenol is surrounded by several aromatic residues including F102^3.30^, F105^3.33^, F182^ECL2^, F203^5.42^_,_ Y260^6.52^ which were also found by functional analysis of mutant receptors to play a crucial role in agonist binding^47^. Furthermore, the three non-aromatic hydrophobic residues L199^5.38^, L259^6.51^ and V277^7.39^ are contacting the agonist molecule. The hydroxyl group in isoeugenol forms an H-bond with Y260^6.52^, which in turn forms an H-bond with E208^5.47^ and water mediated hydrogen bonding to S113^3.41^. Both Y260^6.52^, E208^5.47^ and S113^3.41^ had been shown elsewhere to be important for Olfr73 activation^47^.

**Fig. 3:**
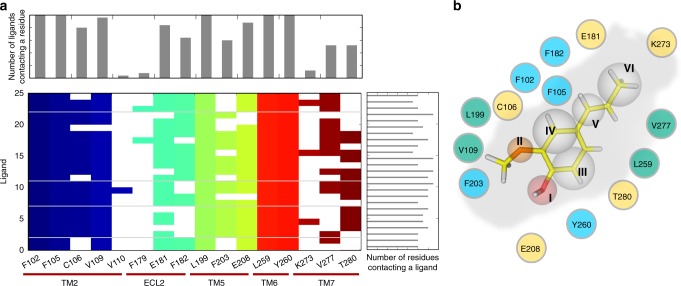
Interaction fingerprints of 25 known Olfr73 agonists grouped in classes 1–5 according to Fig. 4. **a** In the interaction histogram, each contact of a particular residue with the ligand is indicated by a color. The color code distinguishes the residue location in a particular TM helix. Each class of compounds is separated by a horizontal gray line. **b** The pharmacophore model based on 25 known Olfr73 agonists. As a prototypical example, the position of isoeugenol in the Olfr73 binding pocket showing the interaction fingerprint. Assignments: H-bond donor (I), H-bond acceptor (II), hydrophobic moiety (III, IV, V, VI); polar residues (yellow), aromatic residues (cyan), hydrophobic residues (green)

**Fig. 4:**
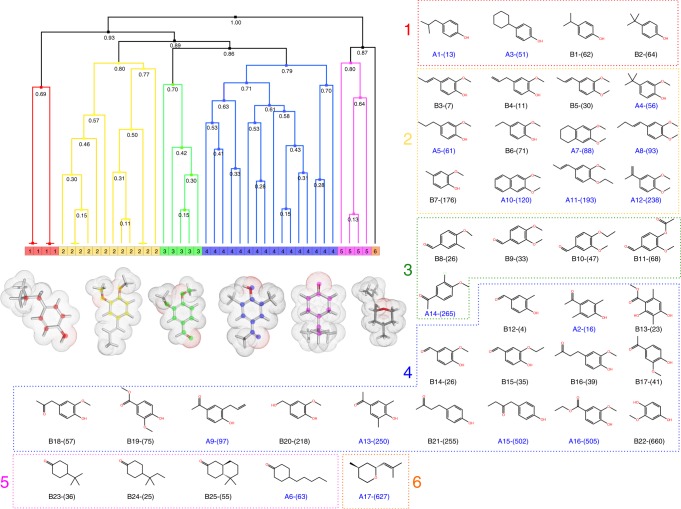
Hierarchical clustering of Olfr73 agonist molecules. Six different classes of agonists are identified (distinguished by a color code) according to their PH4 features. In the Hierarchical diagram, the links between the chemical compounds are represented as branched vertical lines. The height of the lines, coupled with merging distance (numbers showed in each node), indicate the normalized dissimilarity distance between the adjacent compounds. A higher line or a larger merging distance denotes a larger dissimilarity. A typical representative molecular structure of each class is shown below the dendrogram together with their molecular surfaces indicating hydrophobic moieties in grey and polar moieties in red. The commonly shared atoms within a certain class of molecules are labeled with colored dots accordingly. The molecular structures of the six classes of agonists are grouped in boxes. The 17 newly found agonists are represented as A1-A17 in blue. The 25 previously reported agonists are represented as B1-B25 in black. The agonist isoeugenol is B3 and p-isobutylphenol is A1. In all cases the corresponding micromolar EC_50_ values are indicated in brackets. Names of A- and B-compounds are listed in Supplementary Tables 2 and 3

To further validate these observations, we performed an IFP analysis (Fig. 3a), which encodes specific interactions between a particular ligand and specific amino acids in the binding pocket. IFP analyses have been used for computational drug discovery for non-olfactory GPCRs^55^. Here we docked 25 previously reported Olfr73 agonist molecules^16,45^ (Fig. 4) into the binding pocket of Olfr73 and obtained the interaction fingerprints of the different agonists with residues in the binding pocket (Fig. 3a). The IFP analysis showed that all docked agonists could interact with five residues in the receptor’s binding pocket including F102^3.30^, F105^3.33^, L199^5.38^, L259^6.51^ and Y260^6.52^. Furthermore, C106^3.34^(80%), V109^3.37^(96%), E181^ECL2^(80%), F182^ECL2^(64%), F203^5.42^(60%), E208^5.47^(88%), V277^7.39^(52%) and T280^7.42^(52%) are also found frequently (percentage in parentheses) contacting the agonists (Fig. 3a, top histogram). In addition, the three residues V110^3.38^(5%), F179^ECL2^(8%) and K273^7.35^(12%) were found sometimes in contact with the agonists. Each particular ligand was found interacting with at least 80% of all the residues in the binding pocket (Fig. 3a, right histogram). Most of these mentioned residues were found by functional analysis of mutant receptors to play a crucial role in agonist binding^47^.

### Structural characteristics of Olfr73 from molecular dynamics simulations

We modelled the Olfr73 with crystal structures of activated GPCRs. To explore reliable atomic details, we performed 2 × 500 ns all-atom molecular dynamics simulations for both the apo form of the receptor (apo-Olfr73) and the receptor with agonist isoeugenol (iEG-Olfr73) (Fig. 2). The molecular dynamics simulations showed that the volumes of the binding pocket for apo-Olfr73 and iEG-Olfr73 were 190 ± 3 Å^3^ and 220 ± 3 Å^3^, respectively. This is probably because of the induced fit effect (IFD), which has been widely observed in GPCR system and many others^56,57^.

Since the Olfr73 was modelled with low sequence identity templates, it was necessary to restrain the backbone of the modeled OR structure during molecular dynamics simulations to keep correct secondary structure^50,52^. Thus, we added a small force constrain during all our molecular dynamics simulations (see methods section). Transmembrane (TM) movements are a hallmark of GPCR activation. Since the templates used for the molecular dynamics simulations are based on receptors in activated states, the cytoplasmic TM regions of Olfr73 have been kept in the active open conformation by the end of molecular dynamics simulations (Supplementary Fig. 2).

### Virtual agonist screening

We established a refined 3D structural homology model of Olfr73, the agonist-receptor interaction fingerprint and the structural framework explaining the mechanism of receptor activation. To validate these findings, we performed a virtual screen on a large chemical compound library to find new candidates of agonists for Olfr73 (Fig. 1) beyond classical odorant compound libraries, which finally will be tested by cellular functional assays.

First, we evaluated the physicochemical properties of all reported compounds (see Methods section on physical properties filtering) and used them for setting the conditions for an initial filter according to which 312,800 compounds were selected from the initial 1.58 million drug-like compounds of the ZINC library (Supplementary Table 1). For details about this procedure, please see the Methods section on virtual screening).

We applied the next round of selection criteria to our downsized chemical compound library using pharmacophore search (PH4)^58^, a screening method selecting compounds according to their chemical shape (Fig. 3b). The PH4 screen heavily relies on our results obtained from the IFP analysis and the molecular dynamics simulations. According to the molecular dynamics simulations, the oxygen at site I is crucial for agonist binding, forming distinct H-bonds with Y260^6.52^ and E208^5.47^. IFP analysis further confirmed that the interaction with Y260^6.52^ in this position is highly conserved (Fig. 3a). Since the -OH group could be either H-bond donor or H-bond acceptor, it was featured allocated preferentially the PH4 selection filter further downsized the library to 266,000 compounds.

**Fig. 5:**
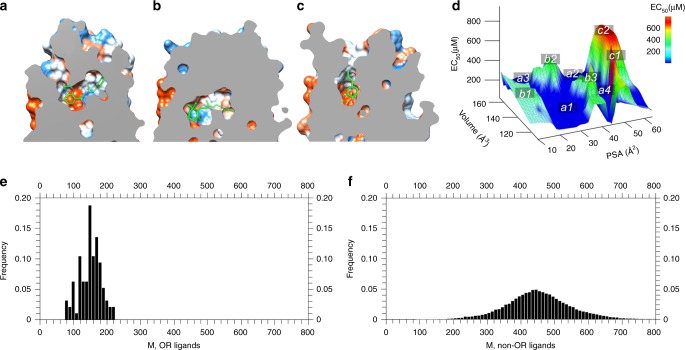
Cross-section through several GPCRs along the membrane normal showing the vertical part of the ligand-binding pocket of (**a**) A_2A_R in complex with ZMA, (**b**) rhodopsin in complex with retinal, and (**c**) Olfr73 in complex with isobutylphenol. **d** Plot of EC_50_ values versus agonist volumes and agonist polar surface areas (PSA) for Olfr73 based on all reported agonists. Highly potent agonists are located in regions *a1*, *a2*, *a3* and *a4*; agonists with medium potency are in the regions of b1, b2 and b3; agonists with lower potency are found in regions *c1* and *c2*. **e** Molecular mass distribution of OR ligands. **f** Molecular mass distribution of non-olfactory GPCR ligands

Interestingly, the empty ligand-binding pocket of Olfr73 has a volume of 190 Å^3^ and is noticeably smaller than that of other GPCRs including A_2A_R (270 Å^3^)^59^, rhodopsin (260 Å^3^)^11^ (Fig. 5), the 5-HT_1A_^60^ receptor (360 Å^3^), or the μ-opioid receptor^57^ (510 Å^3^), and therefore acts as a size-selection filter for potential binders. This explains why all currently reported agonists of Olfr73 are small (MW = 130–220) and the corresponding EC_50_ values are relatively high, due to the limited interactions in such small binding pocket. On this basis we created a volume counter along the 3D space of the sixteen superimposed ligands further reducing the library of potential Olfr73 binders to 493 compounds. We then continued selection filtering applying first an ionization penalty and then a molecular polarity counter, which narrowed the library further down first to 371 and then to 204 compounds.

Finally, we selected potential agonists from the remaining 204 compounds using quantitative structure–activity relationships (QSAR) based on comparative molecular field analysis (CoMFA) methods^61^. We docked the top 100 ranked compounds by QSAR into the MD refined homology model and found that 64 compounds fitted into the ligand-binding pocket of Olfr73, close to the activation trigger F105^3.33^. However, only 25 out of the 64 selected compounds have been commercially available for testing biological activity.

### Cell-based functional tests

Next, we used the SEAP reporter assay, monitoring changes in cyclic adenosine monophosphate (cAMP) second messenger signalling as a read-out for cellular responses of odorant-induced receptor activation and found ligands capable of activating Olfr73 in Hana3A cells^47^ We tested 25 compounds of the molecules predicted from virtual screening and identified 17 (Fig. 4, blue labelled compounds; Supplementary Table 1 and Supplementary Fig. 3) inducing a noticeable SEAP signal in a concentration-dependent activation of Olfr73. It would be interesting to test by additional experiments whether of the eight compounds, which did not show agonist activity, there are antagonists for Olfr73.

### The diversity of OR agonists

In the following, we used a hierarchical agglomerative clustering method^62^ to classify both the newly found and the previously known Olfr73 agonists based on their PH4 characteristics. As shown in Fig. 4, the 42 compounds can be grouped into 6 different classes. The four agonists of class-1 comprise a common phenol group with bulky hydrophobic groups (cyclohexyl or branched methyl containing alkyl chains) in *para* position. The EC_50_ values of class-1 agonists range from 13 to 64 μM. The agonists of class-2 share a central modified pyrocatechol structure (primarily in form of monometoxy-phenol or dimetoxy-phenon) with an additional linear, branched or cyclic hydrophobic group attached. The EC_50_ values of these agonists range from 7 to 240 μM. The agonists of class-3 contain a central benzaldehyde structure. The *para* positions carry primarily a metoxy- or ethoxy-substitute; the *meta* positions are substituted mostly by methoxy groups or for one case by a methyl group. The EC_50_ of class-3 agonists range from 26 to 270 μM. The 16 agonists of class-4, share a central phenol structure with oxygen carrying groups in the *para* and sometimes also in the ortho position. The class-4 agonists are the most polar ones in our collection; ten of them show EC_50_ values in the range of 4–100 μM, the remaining six have EC_50_ values between 200 and 660 μM. The class-5 agonists are quite different from the initial four classes; they do not contain an aromatic ring but instead carry a central cyclohexanone structure preferentially with a linear or branched alkyl substituent at the *para* position. The four class-5 agonists show EC_50_ values from 36 to 63 μM. Only one agonist is listed in class-6. It is composed of a tetrahydro-2H-pyran structure carrying two hydrophobic substitutions in the ring and has an EC_50_ value of 630 μM.

### Therapeutic potential of the newly discovered agonists

We found p-isobutylphenol (4-isobutylphenol) as the most potent ligand activating Olfr73 in our functional assay (Fig. 4). It is a known degradation product of Ibuprofen which is widely used as analgesic anti-inflammatory drug but p-isobutylphenol has also been shown to exhibit antibiotic activity^63^. The estrogenic activity of the compound 4-cyclohexylphenol has been documented by in-vitro assays^64^. The Olfr73 activating compound 4′-hydroxy-3′,5′-dimethoxyacetophenone (Acetosyringone) has anti-asthmatic and anti-inflammatory properties^65^. And finally, 4′-hydroxypropiophenone is a predicted inhibitor of metalloproteinase 10, which has an active role in lung cancer development (Kiresee et al., 2016). Thus, our results have revealed some insights regarding the potential poly-pharmacological profile of these drugs acting not only on a defined medicinal target but also activating an OR. Similar observations of unintended interactions and activation by medicinal drugs have also been documented for the bitter taste receptor TAS2R14^66^. The complete list of newly discovered compounds can be found in Supplementary Table 2. The full list of reported compounds is in Supplementary Table 3.

### Limited volume of OR binding pocket

ORs in general and Olfr73 in particular show some interesting structural and functional differences to their class A GPCR relatives. Most of the known OR-agonists are smaller in size than the typical agonists of non-olfactory class A GPCRs. Considering a large panel of reported OR ligands^67^ together with the new ligands (105 compounds in total) from this work shows that the molecular mass (M) of OR ligands distribute between 80 and 220 Da (maximum around 150 Da) (Fig. 5e). In contrast, non-olfactory GPCR ligands^68^ (161,083 compounds in total) distribute primarily between 300 and 600 Da (maximum around 450 Da) (Fig. 5f). Additionally, EC_50_ values for OR-agonists are usually much higher than those of the agonists of the non-olfactory class A GPCRs^69^. In our present study, this can be explained by the volume of the ligand-binding pocket of the Olfr73 which in the apo form is considerably smaller than comparable regions of the non-olfactory class A GPCRs reducing the number of interaction points between ligand and receptor (Fig. 5). Obviously, the ligand-binding pocket of a particular receptor acts as a size exclusion filter for potential ligands. To test this hypothesis and to probe the flexibility of the ligand-binding pocket for the Olfr73-agonists, we submitted Olfr73 with bound compounds A1, A2 and A3 (Fig. 4) to additional 2 × 500 ns all-atom molecular dynamics simulations. Similar to the agonist isoeugenol, the volume of the binding pocket of Olfr73 increased from 190 ± 3 Å^3^ in the empty state to 220 ± 3 Å^3^ for A1 (MW = 149) to 225 ± 5 Å^3^ for A2 (MW = 166), and 240 ± 2 Å^3^ for A3 (MW = 171), respectively. In general, larger ligands induce a larger volume increase in the occupied binding pocket^56^. Obviously, the binding pocket of Olfr73 is within a certain range quite flexible and adjusts perfectly to the size of the bound ligand with volume changes between 15 and 25%. We previously made similar observations for non-olfactory class A GPCRs such as the P2Y_1_ receptor^70^ changing the volume of the binding pocket from 230 ± 4 to 280 ± 5 Å^3^ (22% change), the 5-HT_1A_^60^ receptor from 360 ± 5 to 425 ± 3 Å^3^ (18%), the A_2A_R^59^ from 270 ± 2 to 315 ± 4 Å^3^ (17%), and the μ-opioid receptor^57^ from 510 ± 3 to 575 ± 5 Å^3^ (13%). In the next step, we performed an interaction fingerprint analysis for our newly found 17 agonists and compared the outcome with that in Fig. 3 of the 25 known agonists (Supplementary Fig. 4). The IFPs of both sets of agonists are quite similar. Moreover, IFPs indicated that there are only two hydrogen bond interactions between Olfr73 and its agonists, whereas there are much more polar interactions in other GPCRs^71,72^. These results further confirm our conclusions that the volume of Olfr73 is limited which is responsible for the small size of its agonists and weak EC_50_ values.

## Discussion

Olfactory receptors represent almost 50% of the human GPCRs and may have additional physiological and pathological functions in the human body beyond their role in olfaction. A critical step allowing studies of the different functional roles of ORs relies on the discovery of their activating ligands. By employing a combination of homology modelling, interaction fingerprint analysis and molecular dynamics simulations to finally find novel agonists beyond classical odorant compounds by in silico screening a large drug compound library. We find that even drug-like molecules can target Olfr73, and compared their OR-activation mechanism to that mediated by classical odorant molecules.

In summary, our study revealed a structural framework for ligand–receptor interactions. We found that the limited volume of the binding pocket is responsible for the small size and weak EC_50_ values of Olfr73 agonists, which is typical for many ORs. Molecular fingerprint analysis discovered the principal interactions between the agonists and the binding pocket comprising many aromatic and hydrophobic residues. Interestingly, when plotting the EC_50_ values vs. ligand volume and ligand polar surface area (PSA), we identified four favorable regions for the most potent agonists with the following characteristics (Fig. 5d): (*a1*) small volume (120–140 Å^3^) and small PSA (20–35 Å^2^); (*a2*) large volume (140–160 Å^3^) and medium PSA (35–40 Å^2^); (*a3*) large volume (>160 Å^3^) and small PSA (10–20 Å^2^); (*a4*) small volume (120–130 Å^3^) and medium PSA (42–45 Å^2^). Three regions with moderate potencies were also identified: (*b1*) small or medium volume (120–150 Å^3^) and small PSA (10–20 Å^2^); (*b2*) large volume (150–160 Å^3^) and small PSA (20–30 Å^2^); (*b3*) medium volume (130–150 Å^3^) and medium PSA (35–40 Å^2^). Two regions with poor potencies were also found: (*c1*) small volume (<130 Å^3^) and large PSA (45–55 Å^2^); (*c2*) large volume (>145 Å^3^) and large PSA (>50 Å^2^).

Comparing with previous work on olfactory receptors^15,43,73^, we found several new aspects in this area including the structural principles leading to higher EC_50_ values for OR activation and the molecular diversity of ligands and interactions with their OR. In conclusion, molecular dynamics simulations in combination with structure based in silico screening offer a promising way to deorphanize the mammalian OR repertoire and thereby contribute to a better understanding of the molecular basis of ligand–OR interactions in olfactory and non-olfactory processes.

## Methods

### Homology modelling of Olfr73

The initial homology models of Olfr73 were obtained by Modeller 9.10^74^ using the crystal structure of two GPCRs in the active state as templates, β_2_AR (pdb code: 4LDE)^48^ and rhodopsin (pdb: 4BEY)^49^; β_2_AR shares 19% and rhodopsin 16% sequence identity with Olfr73. They share the highest sequence identity with Olfr73. 3D multiple sequence alignments were performed by Promals3D^75^ with default settings and were adjusted manually for properly aligning conserved motifs and disulfide bridges. 25,000 structural models (5 × 5000 with different random seeds) were created from the two template structures for Olfr73 in Modeller with fully annealing protocol, and the optimal model was chosen for further study based on Discrete Optimized Protein Energy (DOPE) score. Models from Modeller were submitted to Rosetta for kinematic loop modelling refinement^76^. Over 20,000 structures for loop region were generated. The loop refinement was done in Rosetta^76^.

### Refinement of structural model and protein-ligand docking

The initial Olfr73 structure models generated from Modeller were optimally aligned with the structure of β_2_AR (pdb code: 4LDE) using OPM (Orientations of Proteins in Membranes) database^77^. The pre-aligned Olfr73 structure models were imported into the Maestro v9.3 program^78^. Hydrogens were added to the structures corresponding to physiological pH 7.0. For details please see our previous work^11^.

### Molecular dynamics simulations

We performed restrained molecular dynamics simulations to achieve local improvement of the homology models^52,79^. Using the g_membed^80^ tool in Gromacs^81,82^, the well-prepared Olfr73 structure model was embedded into a pre-equilibrated lipid bilayer of 1-palmitoyl-2-oleoyl-*sn*-glycero-3-phosphocholine (POPC) solvated in 0.15 M NaCl. All molecular dynamics simulations were performed in Gromacs^81,82^. For details please see our previous work^11,83^.

### Interaction fingerprint analysis and compound clustering

Both the interaction fingerprint (IFP) analysis and compound clustering analysis were performed in Schrodinger software^78^. Each compound was first docked into the binding pocket of Olfr73 in Schrodinger, then the interaction fingerprint analysis was performed using the Canvas module^84^. Interaction fingerprints are calculated as a set of bits for the presence or absence of particular types of interactions between a set of ligands and the residues in the active site of a receptor. The IFP divides protein/ligand interactions into four different types: hydrophobic interaction, ion-lock interaction, and H-bond interactions. The frequency of each interaction was calculated by the sum of contacts over the whole frames during molecular dynamics simulations.

The 3D coordinates of each docked compound were imported into Canvas for compound clustering analysis. Canvas first calculates the pharmacophore features (such as H-bond interaction and hydrophobic contact) of imported compounds, then performs hierarchical agglomerative clustering on a set of structures using a similarity matrix. The Hierarchical clustering was generated in Canvas. Hierarchical clustering is a method of cluster analysis which seeks to build a hierarchy of clusters. The merges and splits are determined in a greedy manner^85^. The results of hierarchical clustering are usually presented in a dendrogram. In order to decide which clusters should be combined or split, a measure of dissimilarity between sets of observations is required. In the hierarchical clustering, this is achieved by use of “Average distance between all inter-cluster pairs” method in Schrodinger software^85^, and the Kelley criterion^86^ was used for the linkage which specifies the dissimilarity of sets as a function of the pairwise distances of observations in the sets.

### Virtual screening

We first considered the physicochemical properties of 25 known agonists of Olfr73^17,47^ such as molecular mass (M), calculated logP (clogP), number of rotatable bonds (nRot) and bond valence. However, we extended the scale of each criterium. For instance, as the molecular mass M of the reported compounds are within 134–218 Da, we used a filter for M of 110–320 Da.

The pharmacophore (PH4) search was performed using the Phase^58^ module in the Schrodinger software. PH4 is a fast and efficient tool for shape-based superposition and similarity searching of pharmacophores. A scoring function rank-orders potential pharmacophores by their performance in virtual screening and ligand alignment. The PH4 models were built according to the results obtained from both IFP analysis and molecular dynamics simulations. A H-bond donor/acceptor featured sphere (site I) was created next to the -OH group of Y260^6.52^. A H-bond acceptor descriptor (site II) was placed next to site I. Additional four hydrophobic featured spheres (site III, IV, V and VI) were created according to the IFP analysis.

On the basis of the outcome of the PH4 search, we created a volume counter (230 Å^3^) along the 3D space of the 25 docked ligands to further reduce the compound library. Then we continued the selection filtering applying first an ionization penalty and then a molecular polarity counter. All these steps were performed in the Schrodinger software package.

We selected potential agonists from the remaining compound library using quantitative structure–activity relationships (QSAR) based on comparative molecular field analysis (CoMFA) methods in SYBYL-X 1.3^87^ software. CoMFA considers steric and electrostatic interactions, which would block ligand–receptor interations^88^. As a result, each molecule is located within a three-dimensional grid of defined dimensions. A probe calculates the energy within the bound molecule and neighboring residues of the receptor, in all directions over the entire grid yielding thousands of interactions^88^. Here we used the 25 previously reported^47^ active compounds as training sets. Good correlations (*R*^2^ = 0.73) were obtained between reported experimental results from functional assays and QSAR predicted ligands.

### Cell Culture and Transfection

As described in detail elsewhere^16,45,47^ HEK293T-derived Hana3A cells (provided by Prof. Matsunami, Duke University, USA) were grown in DMEM/F12 medium (Invitrogen, Netherlands) supplemented with 10% fetal calf serum (FCS) (Invitrogen, Netherlands), maintained under selective conditions with 1 μg/ml of puromycin (Sigma, Switzerland) and kept in the incubator at 37 °C and 5% CO_2_. Cells were transfected with plasmid DNA using Lipofectamine 2000 (Invitrogen, Netherlands).

### Quantification of OR responses by SEAP reporter assay

The assays were performed as described in detail elsewhere^16,45,47^ twenty hours before transfection Hana3A cells were seeded into 96 wells (Greiner, Germany) at a concentration of 3.5 × 10^6^ cells per ml of medium. 75 ng of pRTP1S, 150 ng of the cAMP response element fused to the secreted alkaline phosphatase (pCRE-SEAP)^89^ and either 75 ng of pOlfr73^47^ or 75 ng of calf thymus DNA, used as a control, were co-transfected. Compounds to be tested (Sigma, Switzerland) were diluted in DMEM/F12 without FCS and added to the cells 7 h after transfection. Cells were incubated for 16 h at 37 °C in the incubator. For the SEAP reporter assay, the culture medium was mixed with an equal volume of 1 M diethanolamine-bicarbonate, pH 9.8, containing 20 mM para-nitrophenyl-phosphate (pNPP) (Sigma, Switzerland) and 1 mM MgCl_2_ (Sigma, Switzerland). Absorbance was measured at 410 nm using a multiwell absorbance plate reader (Molecular Devices, USA) at 1- to 4-min intervals for a period of 5 min to determine SEAP expression dependent pNPP hydrolysis rates (A_410_ /min). EC_50_ values were determined from dose-response curves fitting experimental data with the Hill equation using IGOR Pro software (WaveMetrics):

$f\left( x \right) = \frac{{f({\rm{max}})}}{{1 + \left( {\frac{{{\rm{EC}}_{50}}}{x}} \right)^n}}\,$*f(x)* is the background-corrected response signal at concentration *x* of the investigated ligand, *f*(max) the maximal, background-corrected amplitude of the response signal, EC_50_ the half maximal effective ligand concentration, and *n* the Hill coefficient. Experiments were performed in triplicate.

### Reporting Summary

Further information on experimental design is available in the Nature Research Reporting Summary linked to this article.

## Supplementary information

Reporting Summary

SUPPLEMENTAL MATERIAL

## Data Availability

The datasets generated during and/or analysed during the current study are available from the corresponding author on reasonable request.
