# Supplementary material for: Computational modeling of the olfactory receptor Olfr73 suggests a molecular basis for low potency of olfactory receptor-activating compounds
Source: Commun Biol. 2019 Apr 23;2:141. doi: 10.1038/s42003-019-0384-8 (PMC6478719; doi:10.1038/s42003-019-0384-8)
Supplement: Supplementary file 2 — SUPPLEMENTAL MATERIAL [file 42003_2019_384_MOESM2_ESM.pdf]

Supplementary Table 1. Characteristics of 25 known agonist molecules and the range used for filtering.

	M	clog P	nBond	nrBond
**Min.**	134	0.9	18	0
**Max.**	218	3.9	39	4
**Range**	110-320	0.6-4.2	16-50	0-10

M: molecular mass
Clog P: calculated log P.
nBond: number of covalent bonds
nrBond: number of rotatable bonds
Min.: the lowest limit found in reported molecules
Max.: the upper limit found in reported molecules
Range: Range of characteristics used for screening filter.

Supplementary Table 2. EC_{50} values obtained from cellular assays for the 25 screened candidates.

ID	Compounds	EC_{50} (μM)
A1	p-isobutylphenol	12.9±0.9
A2	2',5'-dimethyl-4'hydroxyacetophenone	16.0±1.5
A3	4-cydohexyl-phenol	50.8±0.5
A4	4-tert-butyl-2-methoxyphenol	55.6±0.7
A5	dihydroeugenol	61.0±6.9
A6	4-pentylcyclohexanone	63.1±1.5
A7	6,7-dimethoxy-1,2,3,4-tetrahydronaphthalene	88.4±1.3
A8	(E)-4-(but-1-en-1-yl)-1,2-dimethoxybenzene	92.5±0.9
A9	1-(3-allyl-4-hydroxyphenyl)-ethanone	96.9±1.7
A10	2,3-dimethoxynaphthalene	120±1
A11	isoeugenylethylether	193±3
A12	1,2-dimethoxy-4-(1-methylethenyl)-benzene	238±3
A13	4-hydroxy-3,5-dimethylacetophenone	250±3
A14	3-chloro-4-methoxyacetophenone	265±3
A15	4-hydroxypropiophenone	502±315
A16	ethylvanillate	505±109
A17	(2R,4R)-4-methyl-2-(2-methylprop-1-en-1-yl)-tetrahydro-2H-pyran	627±62
A18	6-methoxy-1-tetralone	n.a.
A19	4-tert-butyl-2-methylphenol	n.a.
A20	2,4-diisopropylphenol	n.a.
A21	4'-hydroxybutyrophenone	n.a.
A22	3,4-dimethoxyphenylthiocyanate	n.a.
A23	4-terbutylanisole	n.a.
A24	1-isopropyl-2,3-dimethoxybenzene	n.a.
A25	4'-methoxybutyrophenone	n.a.

n.a: no active response.

Supplementary Table 3. Previously reported compounds shown in Figure 4.

ID	Compounds
B1	4-isopropylphenol
B2	4-(tert-butyl)-phenol
B3	(E)-2-methoxy-4-(prop-1-en-1-yl)-phenol
B4	4-allyl-2-methoxyphenol
B5	(E)-1,2-dimethoxy-4-(prop-1-en-1-yl)-benzene
B6	4-ethyl-1,2-dimethoxybenzene
B7	1,2-dimethoxy-4-methylbenzene
B8	3-methoxy-4-methylbenzaldehyde
B9	3,4-dimethoxybenzaldehyde
B10	3-ethoxy-4-methoxybenzaldehyde
B11	4-formyl-2-methoxyphenyl acetate
B12	4-hydroxy-3-methylbenzaldehyde
B13	methyl-2,4-dihydroxy-3,6-dimethylbenzoate
B14	4-hydroxy-3-methoxybenzaldehyde
B15	3-ethoxy-4-hydroxybenzaldehyde
B16	4-(3-ethoxy-4-hydroxyphenyl)-butan-2-one
B17	1-(3-hydroxy-4-methoxyphenyl)-ethan-1-one
B18	1-(4-hydroxy-3-methoxyphenyl)-ethan-1-one
B19	methyl-3-hydroxy-4-methoxybenzoate
B20	4-(hydroxymethyl)-2-methoxyphenol
B21	4-(4-hydroxyphenyl)-butan-2-one
B22	2-methoxybenzene-1,4-diol
B23	4-(tert-butyl)-cyclohexan-1-one
B24	4-(tert-pentyl)-cyclohexan-1-one
B25	(4aR,8aS)-5,5-dimethyloctahydronaphthalen-1-(2H)-one

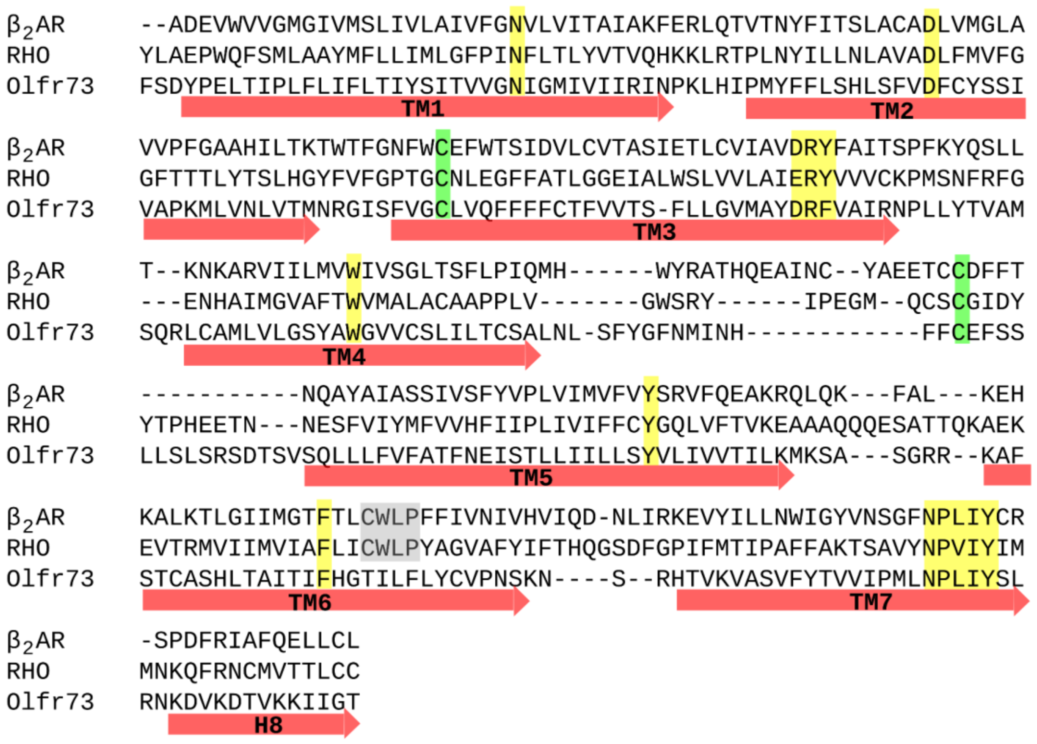

Supplementary Figure 1. 3D sequence alignments of β_2AR (pdb code: 4LDE), rhodopsin (RHO) (pdb: 4BEY) and Olfr73. Red arrows: TM helices; yellow: highly conserved residues or motifs; green: conserved disulphide bridges; gray: CWxP motif.

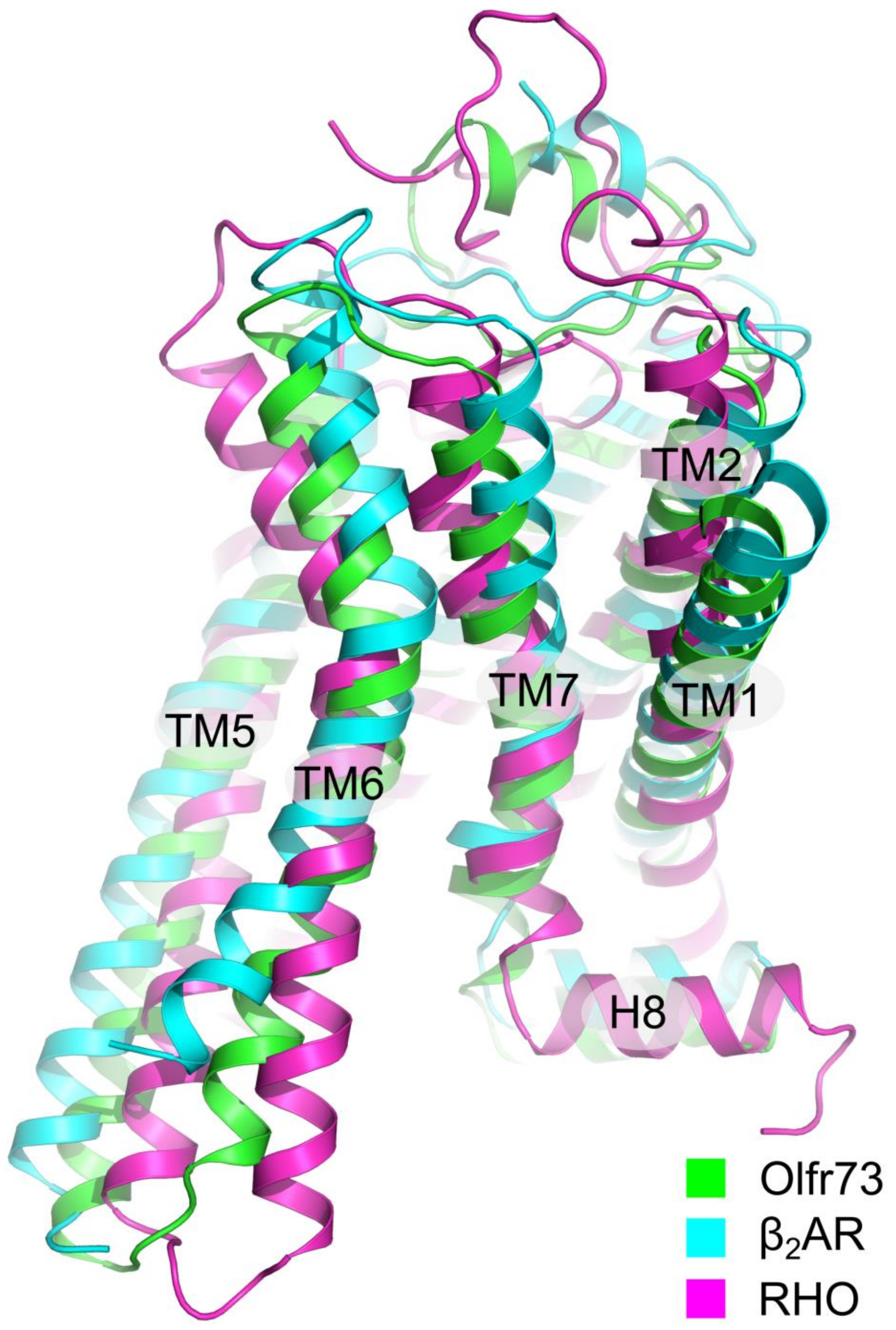

Supplementary Figure 2. The superimposed structures of Olfr73 (green), β_2AR (cyan) and rhodopsin (purple). All structures are in the active states with TM5 and TM6 noticeably outward.

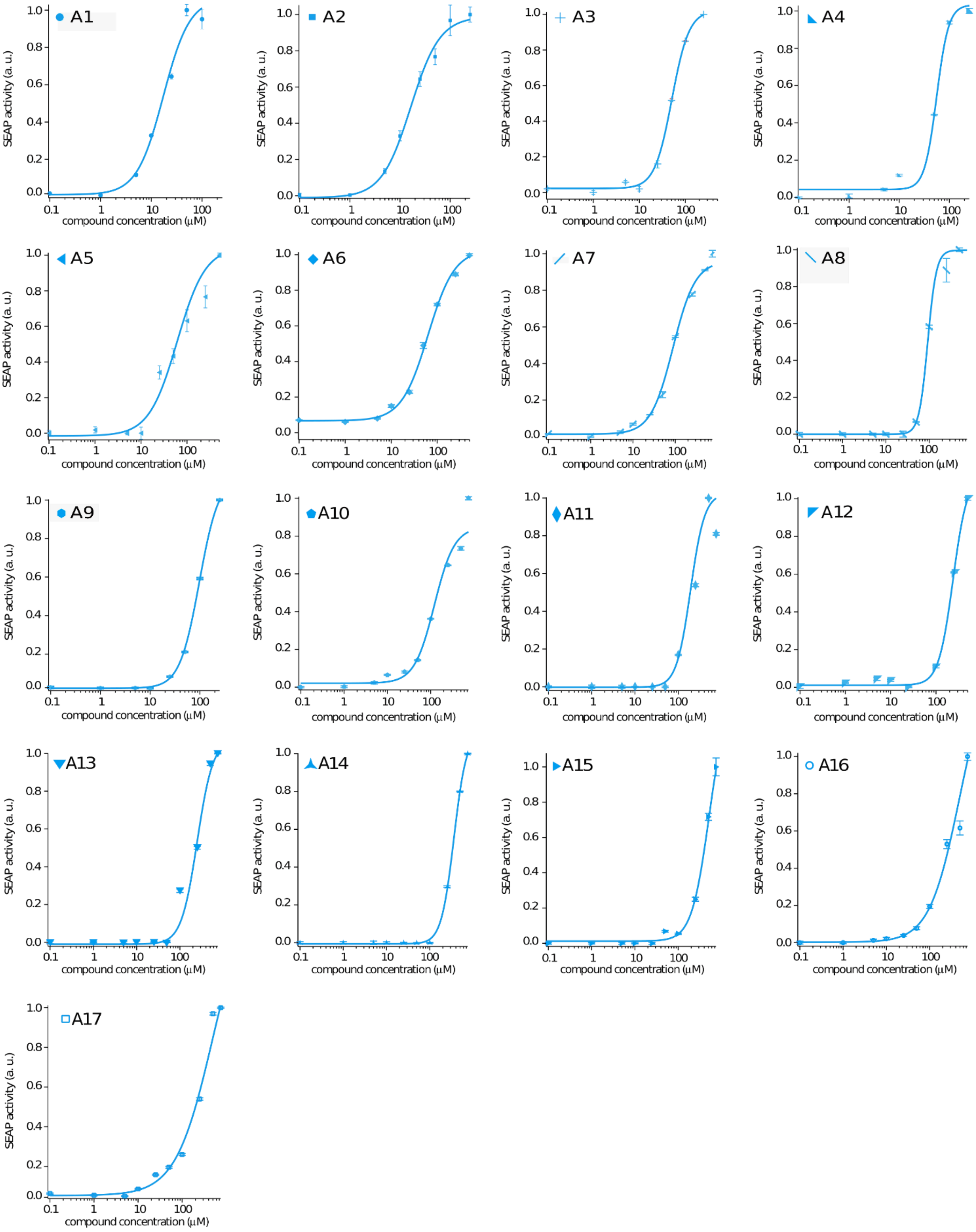

Supplementary Figure 3. Dose-response curves for Olfr73-activating compounds obtained from cellular assays. Numbers according to Table S2.

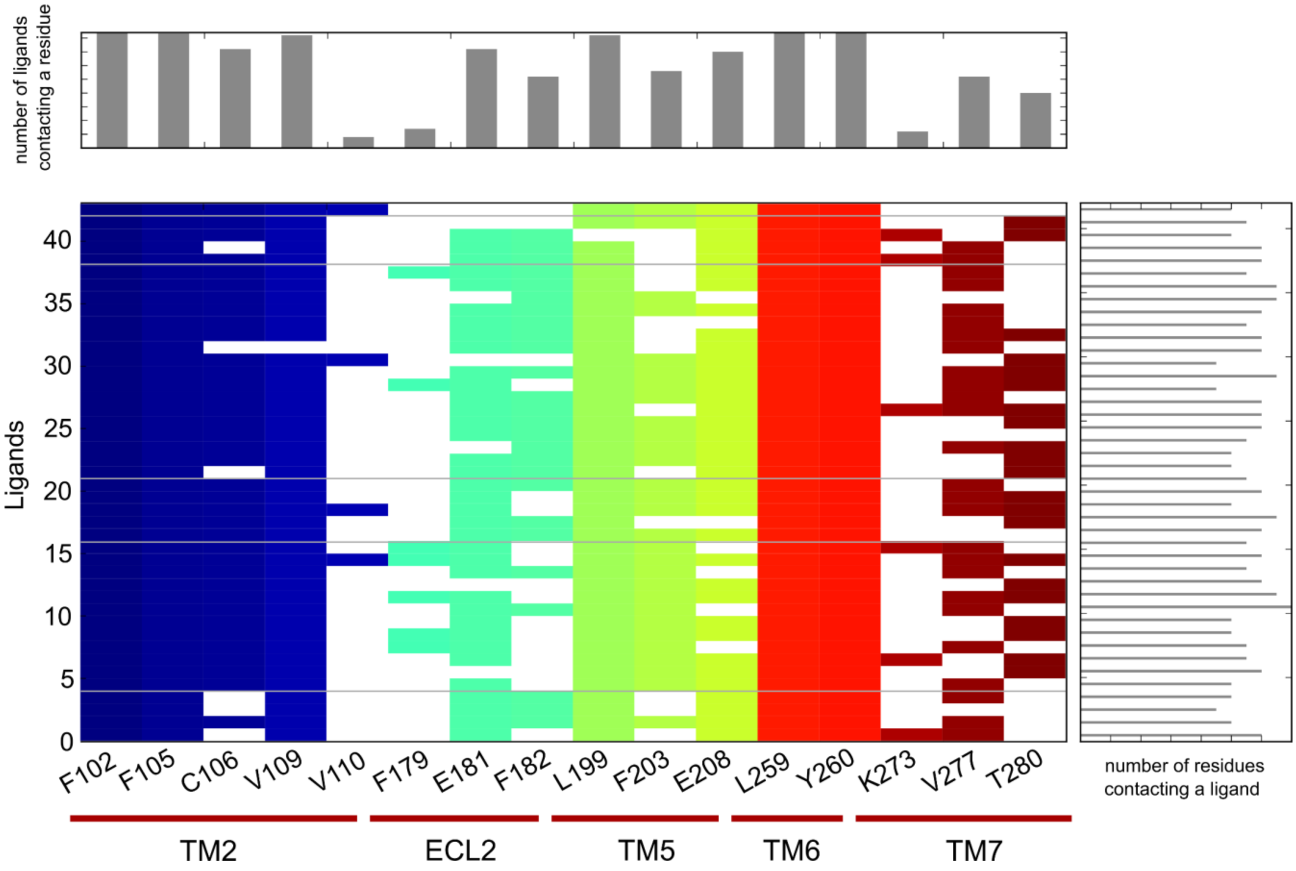

Supplementary Figure 4. The interaction fingerprint of all 42 investigated compounds. The six different classes of agonists are separated by horizontal gray lines according to Fig. 4 (compounds counted consecutively).
